# Sex differences in spiders: from phenotype to genomics

**DOI:** 10.1007/s00427-020-00657-6

**Published:** 2020-02-12

**Authors:** Mathilde Cordellier, Jutta M. Schneider, Gabriele Uhl, Nico Posnien

**Affiliations:** 1 Department of Biology, Institute of Zoology, Universität Hamburg, Martin-Luther-King Platz 3, 20146 Hamburg, Germany; 2 Zoological Institute and Museum, Research Group General and Systematic Zoology, Universität Greifswald, Loitzer Straße 26, 17489 Greifswald, Germany; 3 Department of Developmental Biology, Göttingen Center for Molecular Biosciences (GZMB), University Göttingen, Justus-von-Liebig-Weg 11, 37077 Göttingen, Germany

**Keywords:** Sexual dimorphism, Development, Sex determination, Araneidae, Sexual organs

## Abstract

Sexual reproduction is pervasive in animals and has led to the evolution of sexual dimorphism. In most animals, males and females show marked differences in primary and secondary sexual traits. The formation of sex-specific organs and eventually sex-specific behaviors is defined during the development of an organism. Sex determination processes have been extensively studied in a few well-established model organisms. While some key molecular regulators are conserved across animals, the initiation of sex determination is highly diverse. To reveal the mechanisms underlying the development of sexual dimorphism and to identify the evolutionary forces driving the evolution of different sexes, sex determination mechanisms must thus be studied in detail in many different animal species beyond the typical model systems. In this perspective article, we argue that spiders represent an excellent group of animals in which to study sex determination mechanisms. We show that spiders are sexually dimorphic in various morphological, behavioral, and life history traits. The availability of an increasing number of genomic and transcriptomic resources and functional tools provides a great starting point to scrutinize the extensive sexual dimorphism present in spiders on a mechanistic level. We provide an overview of the current knowledge of sex determination in spiders and propose approaches to reveal the molecular and genetic underpinnings of sexual dimorphism in these exciting animals.

## Introduction

The transfer of genetic information from generation to generation is a key prerequisite for life on earth. Two main strategies have been established: during asexual reproduction, one individual passes its entire genetic information on, for instance by simple cell divisions in bacteria, budding in yeast, fragmentation in annelids or sea stars and vegetative propagation in plants. In contrast, sexual reproduction requires the combination of the genetic material of two individuals. This is achieved by the generation of haploid gametes, which can be morphologically identical (isogamy) or distinct (anisogamy). Most animals are anisogamic with small male gametes and much larger female gametes. The gametes are produced in male- (e.g., testes) and female-specific (e.g., ovaries) organs. Anisogamy sets the stage for sexual selection since small male gametes can be produced in large number whereas gamete numbers are more limited in case of larger eggs. All else being equal, anisogamy will lead to males competing for access to females. Additionally, the need to transfer the male gamete to the female gamete often resulted in the formation of special organs in both sexes such as sperm transfer and sperm storage organs. These sexually dimorphic organs for gamete formation and transfer are often the most obvious phenotypic manifestations of sexual differences (Bell 1982).

While it is well accepted that sexual selection is a major driver for the evolution of sexually reproducing organisms, it is less well established why anisogamy and thus sexual dimorphisms are pervasive in animals. The trade-off hypothesis argues that the separation of sexes is of major advantage because costs can be efficiently allocated between sexes. Male gametes for instance are often optimized to be highly mobile, while female gametes are often optimized to nurture the zygote after fertilization (Bulmer and Parker 2002; Parker et al. 1972). A second main hypothesis argues that the separation of sexes facilitates the avoidance of self-fertilization, thereby maintaining genetic variation (Charlesworth and Willis 2009). Since both hypotheses are supported by empirical data (reviewed in Bachtrog et al. 2014), it remains a major challenge to reveal the exact forces driving anisogamy and sexual dimorphisms.

Sexual reproduction is costly for an organism. It is, for instance, time- and energy- consuming to search for or to attract mating partners, and the generation of haploid gametes and the development of specialized organs to host and transfer gametes is a major investment for an organism. Therefore, organisms that reproduce asexually can propagate much faster and should outperform sexually reproducing organisms (Bell 1982). However, only very few animal and plant species reproduce predominantly asexually, suggesting that the investment in sexual reproduction must provide a selective advantage (e.g., Otto 2009). An obvious advantage of combining different genetic variants segregating in a population is that deleterious mutations cannot accumulate over time (Agrawal 2001; Hussin et al. 2015). Additionally, in the light of competitiveness among males, where a single male can in principle fertilize many females while others do not mate at all, sexual reproduction facilitates sexual selection because individuals can choose with whom to mate. Therefore, in addition to primary sexually dimorphic traits many secondary phenotypic differences, such as massive horns in males of some ungulates or beetles, long noses in males of the proboscis monkeys and colorful structures that are used for display in many birds and jumping spiders evolved between sexes (Andersson 1994; Huber 2005). In addition to morphological traits, sexes differ extensively in behavioral and life history traits. Males may grow much larger than females if body size promises high mating success (reviewed in Fairbairn 2013) as known for many mammals that defend harems, such as elephant seals. However, in many arthropods, females grow larger than males and this is often explained by female fecundity that positively correlates with size (Blanckenhorn 2005). Males often show elaborate behavioral displays to impress females perhaps most spectacularly in birds of paradise (Ligon et al. 2018). Such traits evolved under sexual selection because they entail advantages in male-male competition and/or female choice (Andersson 1994).

To understand the evolution of sex and sexual dimorphism more mechanistically, we need to gain comprehensive insights into the molecular and genetic mechanisms underlying sex determination. The analysis of sex determination mechanisms in a few genetically established model systems such as *Drosophila melanogaster* and *Caenorhabditis elegans* revealed conserved as well as highly diverse aspects of this process. It has for instance been shown that the expression of genes coding for Doublesex and Male-abnormal-3 Related Transcription factors (Dmrt) seems to play central roles in sex determination in many animals studied to date (Bachtrog et al. 2014; Bopp et al. 2014; Cline and Meyer 1996; Gamble and Zarkower 2012). In contrast, the processes leading to the expression of *Dmrt* genes are highly variable, as exemplified by the fact that the male determining factor of the house fly *Musca domestica* can be located on any chromosome in different populations (Hamm et al. 2015). In light of the diversity of sex determination systems, many more taxa need to be studied to understand how sex determination mechanisms and sexual dimorphisms evolve (Gamble and Zarkower 2012; Zuk et al. 2014). This is even more relevant since some of the species in which sex determination has been intensively studied with respect to genetics and development show only modest sexual dimorphism.

**Fig. 1:**
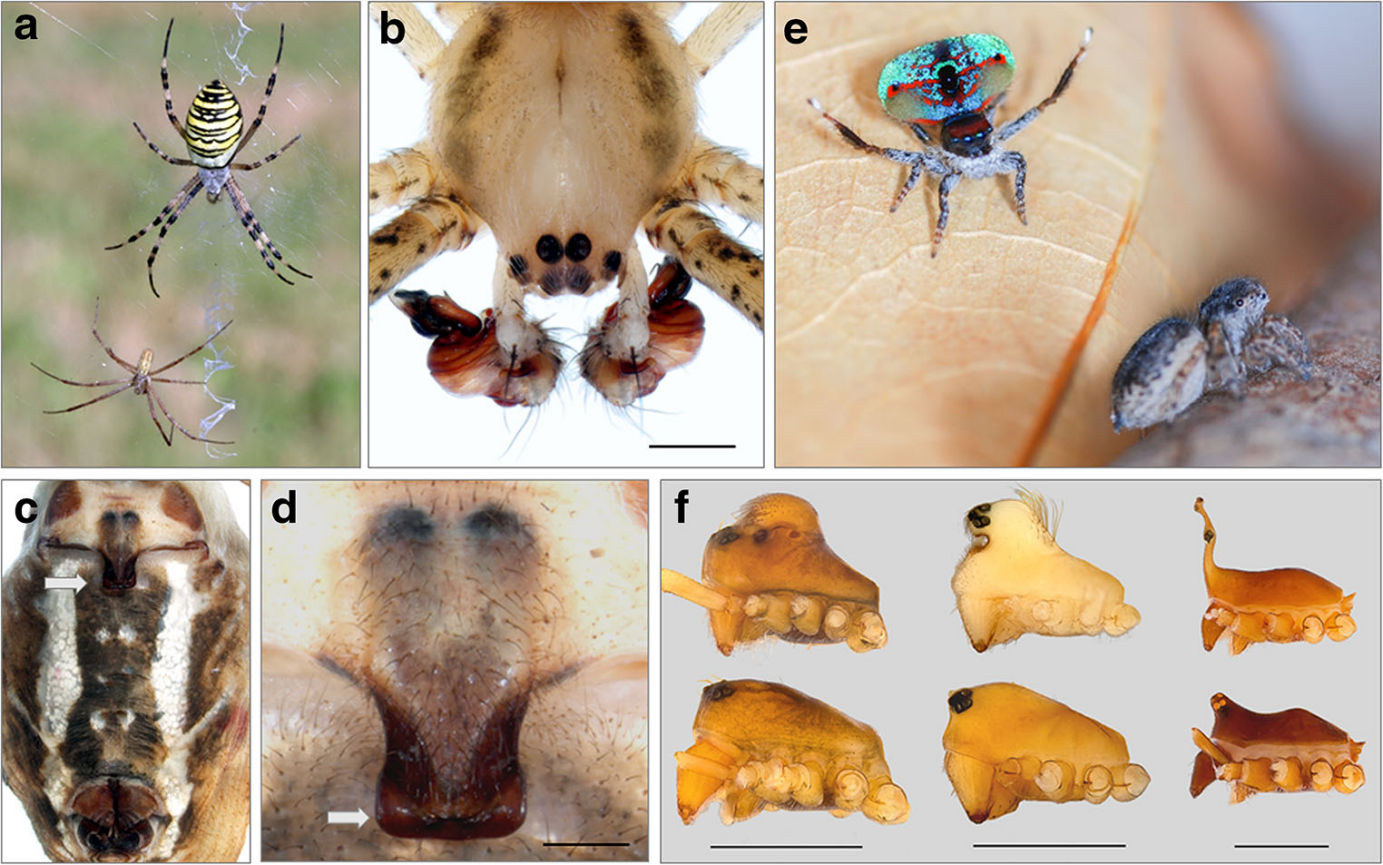
Dimorphism in primary and secondary sexual traits (size, shape, and color) in selected spider species. (a) *Argiope bruennichi* sexual dimorphism in body size and shape, female above, male below (phot. S. Nessler). (b) *A. bruennichi*, close-up of male secondary copulatory organs (pedipalps) (phot. S.-W. Lin). (c) *A. bruennichi* female ventral side with genital region (arrow: scape) (phot. S.-W. Lin). (d) *A. bruennichi*, close-up of scape (arrow) on the female genital opening (phot. S.-W. Lin). (e) *Maratus mungaich*, dimorphism in shape and color of male (above) and female (below) (phot. A. Aceves-Aparicio). (f) Prosoma of male (upper) and female (lower) erigonines, lateral view. From left to right: *Hybauchenidium aquilonare*, *Shaanxinus hirticephalus, Walckenaeria acuminata* (phot. S.-W. Lin & L. Lopardo). Scale bars: b, d, 0.5 mm; f, 1 mm

In this perspective article, we argue that spiders, a diverse group of predators with more than 48,000 described species (World Spider Catalogue, 2019, last accessed January 2020), are an excellent group in which to study various aspects of sexual dimorphism, sexual reproduction, and sex determination because they comprise the most remarkable sexual size dimorphism among terrestrial animals as well as highly elaborated sexual signals comparable to those of birds of paradise (Fig. 1 a, e, f). We first outline the extent of sexual dimorphism in morphology, behavior, and life history in araneomorph spiders. Although morphological and behavioral specializations of the sexes are striking, we know very little about the genetic and genomic background of sex differences in adults and how they are defined during development. We argue that spiders are an ideal group for the study of sex differences in development across the entire life span, from the fertilized egg through to maturation and beyond. The time is ripe for exploring these fundamental aspects since genomic resources have become available for an increasing number of spider species (Garb et al. 2018). We highlight first results on the genetics and genomics of spider sex determination and propose potential starting points to elucidate the genomic basis of the multifaceted sexual dimorphism in spiders.

## Phenotypic manifestation of sexual dimorphism in spiders

### Morphology

Adult spiders exhibit extensive sexual dimorphism in external morphology. Males can be much smaller than females like in many orb-weaving spiders (Fig. 1a) or much more colorful like in many jumping spiders (Fig. 1e). The most reliable way to distinguish the sexes is by the shape of their paired pedipalps which are situated anterior to the four pairs of walking legs. While they are leg-like structures in females that are used to probe substrate and prey, they serve as secondary sexual organs in males that often have the appearance of enlarged boxing gloves (Fig. 1b) (Foelix 2011). Sperm is produced in paired testes inside the opisthosoma but transferred and stored until mating in specific organs at the tips of the male pedipalps. These palpal organs can be simple and tear-shaped or comprised of a complex set of sclerites and inflatable membranes (Fig. 1b). Palpal organs are generally highly species-specific and used for species identification (Foelix 2011). When the male encounters a receptive female, the palpal organ is inflated by hydraulic pressure which causes considerable changes in the relative positions of the sclerites. Some sclerites are used as locking devices that attach to specific structures on the female’s genital region, and some are introduced into the female genital openings (Huber 1995; Uhl et al. 2007). In many species, the genital opening of the females is characterized by species-specific sclerotized structures that surround or overlap the paired copulatory ducts, as in many entelgyne spiders (Fig. 1 c, d). When the sperm is discharged into the sperm storage sites of the female, the sperm are stored in an encapsulated state in these spermathecae until egg laying (Vöcking et al. 2013). Females produce eggs in paired ovaries (Foelix 2011). During the egg-laying process, the stored sperm is activated in the spermathecae and meets the eggs in the oviduct or genital atrium. In spiders, the number and arrangement of the spermathecae and their connected ducts are as highly diverse and species-specific as are the copulatory mechanisms. Consequently, there are manifold possibilities of interactions between male and female genital structures that allow e.g. separate storage of different ejaculates, differential sperm activation, removal of rival sperm, or plugging of female ducts to prevent the female from remating (e.g., Herberstein et al. 2011; Schneider and Andrade 2011; Uhl et al. 2010). The genital morphology of spiders therefore opens up many ways for females to control paternity and for males to secure paternity—resulting in sexual conflicts.

Adult male and female spiders generally differ in body weight, but they can be of similar size and shape or differ markedly, depending on species. In similarly-sized species, such as most cursorial spiders, adult females differ from males - not only in the form of the pedipalps but also in body shape- mainly because females build up resources in the opisthosoma for egg production (but see Fernández-Montraveta and Marugán-Lobón 2017). Strong sexual dimorphism in size with large females and small males occurs in many orb web spiders (Foellmer and Moya-Larano 2007; Robinson and Robinson 1973). The most extreme case of reversed sexual size dimorphism among terrestrial animals can be found in the family Nephilidae (Kuntner and Coddington 2020), where tiny males enter the web of a giant female for mating, which then - more often than not - ends with him being a mate and a meal. Spider species with larger males than females are rare (e.g., Lycosidae: Aisenberg et al. 2007; Pholcidae: Huber et al. 2013; *Argyroneta aquatica*: Schütz and Taborsky 2003). Apart from differences in size and overall body shape, males of many species may carry ornaments, such as intriguing color patterns (Stavenga et al. 2016), enlarged legs that are equipped with bristles (Stratton 2005), or even eye-stalks or bizarre protrusions on their prosoma, the front body part (Hormiga 2000; Huber and Nuñeza 2015; Michalik and Uhl 2011; Vanacker et al. 2003). In fact, males and females of the same species were described as different taxa due to their extreme differences in size, shape, and color (e.g., Kuntner et al. 2012). Generally, morphological sex differences do not appear until one or two molts before maturation (see below).

After the molt to maturity, males of the web-building species change their sedentary lifestyle to start searching for mates. As an adaptation to this transition they reduce the number of silk glands and spinnerets (and references in Correa-Garhwal et al. 2017; Kovoor and Peters 1988). Mainly those silk glands seem to be left that produce the silken dragline, whereas all silk glands and spinnerets remain active in the females. As a consequence, males lose the ability to spin a capture web after the final molt; if males require food during their adult phase, kleptoparasitism in the web of the females is the only foraging option (Foelix 2011; Martišová et al. 2009). However, recent gene expression studies in *L. geometricus* and *L. hesperus* showed that silk gene expression in males was more diverse than expected if only dragline silk were produced. Males showed high expression of minor ampullate silk genes which are perhaps used to spin sperm webs or to produce silk for dispersal, so-called ballooning events (Correa-Garhwal et al. 2017).

### Behavior

Spiders are known for their diverse hunting strategies, with or without silk, that range from highly specialized pheromone traps by the bolas spider (Eberhard 1980) or spiders that mimic ants, to exquisite silken structures designed to capture cursorial or aerial prey (Pekár et al. 2017). However, these behaviors are not known to show much sexual dimorphism until the adult stage. Besides the obvious morphological sexual dimorphisms, equally fascinating sex differences can be observed in the mating behavior of spiders.

### Mating behavior

When it comes to spiders mating, the phenomenon of sexual cannibalism is likely the most widely known behavior that shows a clear sex difference, since it is generally the male that is killed by the female (Elgar and Schneider 2004) although exceptions do occur (Sentenská and Pekár 2013; Sentenská and Pekár 2014). However, there are more interesting sex differences in spider behavior during the entire process - starting with finding a mate. The first fundamental sex difference is that males are generally the searching sex, although searching females were also observed (Aisenberg and González 2011).

The sexes find each other mostly because males find females, but there are differences between the two major life styles in spiders, namely between species that hunt with a web and those that do not. In the latter cursorial spiders, including jumping, wolf, crab, and wandering spiders, male mate search seems to be primarily based on chemical cues on dragline silk of receptive females. Studies on several cursorial spider species have shown that males respond to female draglines and follow them (Anderson and Morse 2001; Beyer et al. 2018; Rypstra et al. 2009). Seismic signaling by males to attract and impress females does also occur. Male wolf spiders drum on dead leaves to attract females, and the drumming rate was found to be a reliable indicator of male condition similar to the vocalization displays of red deer (Kotiaho et al. 1996; Mappes et al. 1996). Web-building females stay in their web while adult males abandon their sit-and-wait life style and actively search for mates. This alteration in life-style comes along with several morphological changes (see above). Other than using draglines as carrier for chemical cues, receptive females of web-building species produce volatile pheromones that males respond to by approaching the web. Sex pheromones are characterized for a handful of species to date (Fischer 2019; Schulz 2004; Uhl 2013).

Mate approach and courtship can be dangerous since spiders are generally cannibalistic (Elgar and Schneider 2004). Many wolf and jumping spiders are known to use multimodal displays, mostly combinations of visual signaling and vibrations (Hebets and Papaj 2005; Herberstein et al. 2014; Uhl and Elias 2011). In web spiders, males mostly use vibratory signals on the web and/or chemical signals to reveal their identity and suppress an aggressive response from the female (Becker et al. 2005; Wignall and Herberstein 2013). Spiders are highly interesting models to investigate the evolution of complex signaling, and there is some excellent research to build upon, both on the production side and on the perception side (Barth 2002).

Once males find a female, regardless of whether they enter a web or not, they face the challenge of male competition. Many studies have investigated pre-mating competition leading to contests and mate guarding (Schneider and Andrade 2011). More indirect male behaviors that occur in a female web reduce male-male competition, for example in the sheet web spiders *Nereine* (previously *Linyphia*) *litigiosa* and *Linyphia triangularis*. In theses species, males wrap up and remove large parts of the web of the females and thereby reduce the amount of pheromone transmitted into the air and consequently the probability that a rival finds the female (Schulz and Toft 1993; Watson 1986, 1990, 1991). Excluding rivals is highly beneficial for males only if females are polyandrous, which appears to be common in spiders.

As a result of anisogamy, evolutionary interests of males and females may not align, for example concerning mating rate (Arnqvist and Rowe 2005), inducing a sexually antagonistic coevolution (Rice 1996). A conflict of interest is particularly evident in sexually cannibalistic spiders and has been studied in detail in species characterized by a monogynous mating system (Schneider and Fromhage 2010). Monogyny evolved several times independently in spiders and is associated with extreme sexual size dimorphism. Conflict arises between males that invest maximally in monopolizing paternity with a single female while the female appears to counter this strategy by cannibalizing the male before he achieves this goal. A resulting antagonistic coevolution has led to fascinating behaviors in both sexes, and their convergent evolution provides ideal conditions to study the evolution of extreme mating strategies (Schneider 2014).

Theory has shown that a monogynous strategy evolves under a male-biased sex ratio and is only stable if the mating investment will yield a higher than average paternity share (Fromhage and Schneider 2012). Males achieve this for example by breaking off pieces of their genitalia that will be used to plug the genital openings of females and prevent future rivals from gaining paternity (Fromhage and Schneider 2005; Nessler et al. 2007; Uhl et al. 2010). In many species that show an extreme sexual size dimorphism, males are cannibalized by the females during mating—often the first mating—or die spontaneously after having used both palps. The most spectacular and best-known mating behavior is perhaps the male somersault in *Latrodectus hasselti* where the male abdomen comes to rest on the female chelicerae during copulation (Andrade 1996). The female starts feeding on the male but he has pulled all important organs to the front part of the abdomen (Andrade et al. 2005). Thereby males survive their first copulation and can inseminate both spermathecae of a female. Males have also evolved curious ways to circumvent female control of mating by immature mating in *L. hasselti* and *L. geometricus* (Biaggio et al. 2016) and by mating while the female is molting in *A. bruennichi* (Uhl et al. 2015).

In species in which males survive copulations, they may cohabit with the female after mating which likely has a mate guarding function. Males may also cohabit for other reasons as for example to steal prey from females (kleptoparasitism, e.g., Martišová et al. 2009). Males of *Stegodyphus lineatus* (Eresidae) have been shown even to gain weight during the days of cohabitation; however, females stop renewing their webs if a male is present (Erez et al. 2005). Generally, except for the monogynous mating systems mentioned above, male fitness increases with the number of receptive females he encounters and mates with (Bateman 1948). Accordingly, males should economize on time spent with mate-guarding and stealing prey and continue to search for females. In cursorial species, females cannot easily be defended by males, which should have general implications on the evolution of mating systems. Mating behavior is highly diverse in spiders and many more fascinating behaviors wait to be discovered. Variation occurs not only between spider families or genera but also between closely related species, offering interesting material for evolutionary studies.

### Dispersal behavior

Most spiders show a gregarious phase after hatching that lasts but a few days (Chiara et al. 2019) while it is extended for longer in brood-caring species and permanent in social species. Solitary spiders disperse as small spiderlings via a mechanism called ballooning. The spiders become airborne by releasing silk threads under favorable certain environmental conditions such that the drag will lift them (Cho et al. 2018; Weyman 1993). Ballooning and short distance dispersal can be triggered by density and resource availability (Puzin et al. 2019) and vary individually (Johnson et al. 2015) for example due to paternal effects (Mestre and Bonte 2012). All these factors may differ in relevance between males and females.

In many animals, sexes differ in their dispersal behavior and commonly discussed causes are inbreeding avoidance, local mate competition, or local resource competition (see Li and Kokko 2019 for a recent review). In social *Stegodyphus* spiders, larger and even mature spiders have been observed to balloon (Schneider et al. 2001; Wickler and Seibt 1986). Here, adult females mate within their natal colony and either stay and reproduce or disperse to found new colonies (Lubin and Bilde 2007). Males mate with their sisters leading to high degrees of inbreeding. However, males also show short distance dispersal and enter other colonies (Aviles and Purcell 2012; Settepani et al. 2017; Smith et al. 2016; Lubin et al. 2009; Smith et al. 2016) thus causing gene flow between colonies. While we can directly observe dispersal in large adult *Stegodyphus*, sex differences in species with juvenile dispersal can only be inferred from population genetic studies that cannot easily differentiate between sex-specific dispersal behavior and sex-specific mortality during dispersal. The theoretical predictions for sex-specific dispersal are likely to apply to spiders but empirical tests are rare (Mestre and Bonte 2012). Further, endosymbionts such as *Wolbachia*, *Rickettsia*, or *Spiroplasma* that are known as sex ratio distorters are predicted to favor male dispersal and female philopatry (Bonte et al. 2009).The lack of diagnostic genetic or morphological markers that allow sexing large numbers of spiderlings prevents research on sex differences in spider dispersal.

### Life history

#### Reproductive investment strategies

Males invest their resources into mate searching and male-male competition as well as sperm production while female spiders invest substantially in their offspring. Maternal allocation strategies range from the production of a few, very small eggs followed by intensive brood care to the production of hundreds of eggs that are deposited inside a more or less protective silken cocoon and then left alone (Foelix 2011). Paternal care is extremely rare in spiders (Mora 1990), and most males of web-building spiders are long dead when the females start to lay eggs. Therefore, the sexes differ significantly in reproductive investment in spiders.

#### Plasticity in growth

Spiders are much more flexible in all life-history variables than earlier studies suggested. Indeed, spiders have been recently advocated as ideal model organisms for the study of adaptive plasticity (Andrade 2019). Generally, individuals respond to external cues in all known life-history decisions and show a high degree of plasticity (Andrade 2019).

Maturation time is the final crucial life-history decision each individual has to make (Roff 1992). Early maturation might be problematic for both sexes because mates may not be available yet and maturing too late entails the same risk. Biotic variables such as prey or predator abundance as well as abiotic conditions such as temperature might further punish early or late maturation. Spiders grow discontinuously through molting, a costly and risky process (Foelix 2011). Spiders are soft and cannot move when shedding the old skin and before the new skin has hardened. Hence, the number of molts should be optimized by selection. Species differ in the number of molts required until maturation and the number of molts to reach maturation differs between females and males in many species (Foelix 2011). Evidence is increasing that both instar number and instar duration are plastic traits in both sexes and respond to external conditions such as prey availability and quality, daylength, temperature, and even social cues. Growth studies have shown that females and males respond differently to variation in diet (Neumann et al. 2017; Uhl et al. 2004) suggesting sex-dependent growth strategies and divergent selection pressures on male and female life-history traits (Kleinteich and Schneider 2010; Uhl et al. 2004). Particularly in species with a large sexual size dimorphism, males are affected most strongly by food restriction during early life, while females are generally able to compensate early restrictions if conditions improve later in life. Studies in *Trichonephila senegalensis* have shown that females delay maturation and reach the body size of sisters that were raised under high food conditions (Neumann et al. 2017). Body size and body condition are generally good predictors for fecundity in spiders (Head 1995), and current knowledge supports that females optimize body size if necessary, at the cost of delayed maturation. In contrast, male reproductive success may often depend more strongly on the timing of maturation than on body size, particularly under scramble competition.

The divergent growth patterns of males and females suggest early differences in the processes underlying growth. Sex differences could be caused by differential maternal allocation, positions in the egg-clutch might differ between the sexes, and sibling cannibalism might be sex-specific as well. Regulation of molting including time between molts, number of instars, and weight increase per time likely differ as well. So far, we are lacking the tools to sex early developmental stages and to our knowledge the mechanisms behind growth plasticity are completely unexplored. Trophic eggs are described in some species and there might be competition over such extra resources. Another potential factor that may provide a head start and increases variation in early growth is sibling cannibalism. Cannibalism among siblings in or outside the cocoon has been reported from many species, but it has been intensively studied in only a small number of species. In *Latrodectus hesperus*, for example, it was convincingly shown that hatching asynchrony enhances sibling cannibalism (Johnson et al. 2016).

## Development of sexual dimorphism in spiders

### The subadult instar and sexual maturation

While sexual dimorphisms are obvious in adult traits such as morphology, behavior, and life history, the developmental and molecular mechanisms underlying the formation of sexually dimorphic traits remain largely elusive. In araneomorphs, the first morphological sex differences are apparent in the last instar before sexual maturation (i.e., subadult instar) when the male pedipalps thicken distally. A morphological analysis of bulb development inside the tip of the pedipalp in *P. tepidariorum* suggests that its morphogenesis involves a complex sequence of events (Quade et al. 2019). This work established that the anlagen of the bulb are already present in the distal tip of the pedipalp in the pre-subadult stage, strongly suggesting that male-specific development is initiated much earlier. Interestingly, spermatogenesis already begins during subadult stages in the cellar spider *Pholcus phalangioides* (Michalik and Uhl 2005), and *Loxosceles intermedia* (Margraf et al. 2011) suggesting that the male genital system must develop prior to this stage already. It is likely that also the development of external female genitalia precedes the subadult stage since the epigyne is already visible below the cuticle before the final molt in some spiders (Biaggio et al. 2016, Uhl unpublished). Body shapes can also start to differentiate between sexes throughout the last two instar stages (Uhl unpublished). Based on these observations, the development of sex differences must be initiated prior to the pre-subadult stage in males and females. However, it remains to be established whether the development of primary and secondary sexual traits is a gradual process that is initiated already during embryogenesis or is initiated only few molts before maturation. Hence, a thorough morphological description of organ development is missing that allows identifying crucial stages at which the anatomy diverges between males and females.

### Developmental genetics underlying sex determination

Developmental processes are regulated by the action of developmental gene products. While the genetic underpinnings of early spider embryonic development, such as axis formation (Akiyama-Oda and Oda 2006; Oda et al. 2019; Pechmann et al. 2017), segmentation (Paese et al. 2018; Pechmann et al. 2011; Schönauer et al. 2016; Stollewerk et al. 2003), or visual system development (Samadi et al. 2015; Schomburg et al. 2015), are being revealed these days, the genetic mechanisms regulating sex determination are entirely unclear. In general, the sex in animals can be defined by environmental cues, by genetic factors or a combination thereof. Environmental sex determination can for instance be observed in crocodiles where the temperature during early embryonic development plays a pivotal role (Janzen and Paukstis 1991). A combination of several environmental cues such as photoperiod, food resources, and density has been shown to determine the sex in the branchiopod crustacean *Daphnia magna* (Kato et al. 2011). However, in the majority of animals, sex is determined genetically. Our current understanding of these processes in arthropods is mostly based on detailed genetic analyses in the well-established model system *D. melanogaster* where the sex determination cascade can be subdivided into three main steps (reviewed in Bopp et al. 2014; Cline and Meyer 1996; Gamble and Zarkower 2012; Herpin and Schartl 2015):

1. Instruction: The genomic architecture of an individual provides the first instructive signal. In *Drosophila* the instruction is derived from the interpretation of the proportion of X chromosomes to autosomes. Females have a 1:1 ratio with two X-chromosomes and two copies of each autosome, while males possess a 0.5 ratio. The presence of two X chromosomes in females leads to the expression of the gene *Sex lethal (Sxl)*.
2. Transduction: The activity of the Sxl protein in females results in female-specific splicing of the pre-mRNA of the *transformer (tra)* gene what results in a functional Tra protein. Tra itself is a splicing factor that regulates the female-specific pre-mRNA splicing of the *doublesex*^*F*^*(dsx*^*F*^*)* gene. The male splice variant of *tra* is not functional. This results in the absence of TRA protein, which in turn causes the production of a male-specific splice form of the *Dsx* gene.
3. Execution: *dsx*^*F*^ codes for a transcription factor that activates the expression of genes required for female developmental programs.

In the presence of only one X chromosome in males, no Sxl will be generated resulting in male-specific splicing of *tra* pre-mRNA. A premature stop codon prevents the generation of a functional Tra protein what leads to the formation of a male-specific Dsx^M^ transcription factor, which in turn activates target genes required for male development.

Some of these three common steps underlying the molecular control of sex determination in *D. melanogaster* are highly conserved across different animals*.* For instance, the triumvirate Sxl/Tra/Dsx or their orthologs are pivotal in many cases (Bopp et al. 2014). A database search (OrthoDB and NCBI) in available spider genomes revealed that most spider species possess one or more orthologs of the well-known sex determination genes. Therefore, these genes are an excellent starting point to reveal candidate factors involved in spider sex determination. In the following, we first summarize sparse data on potential genetic mechanisms and we then propose strategies to identify sex determination factors in spiders, starting with the final readout of the sex determination cascade.

### Execution and beyond

**Fig. 2:**
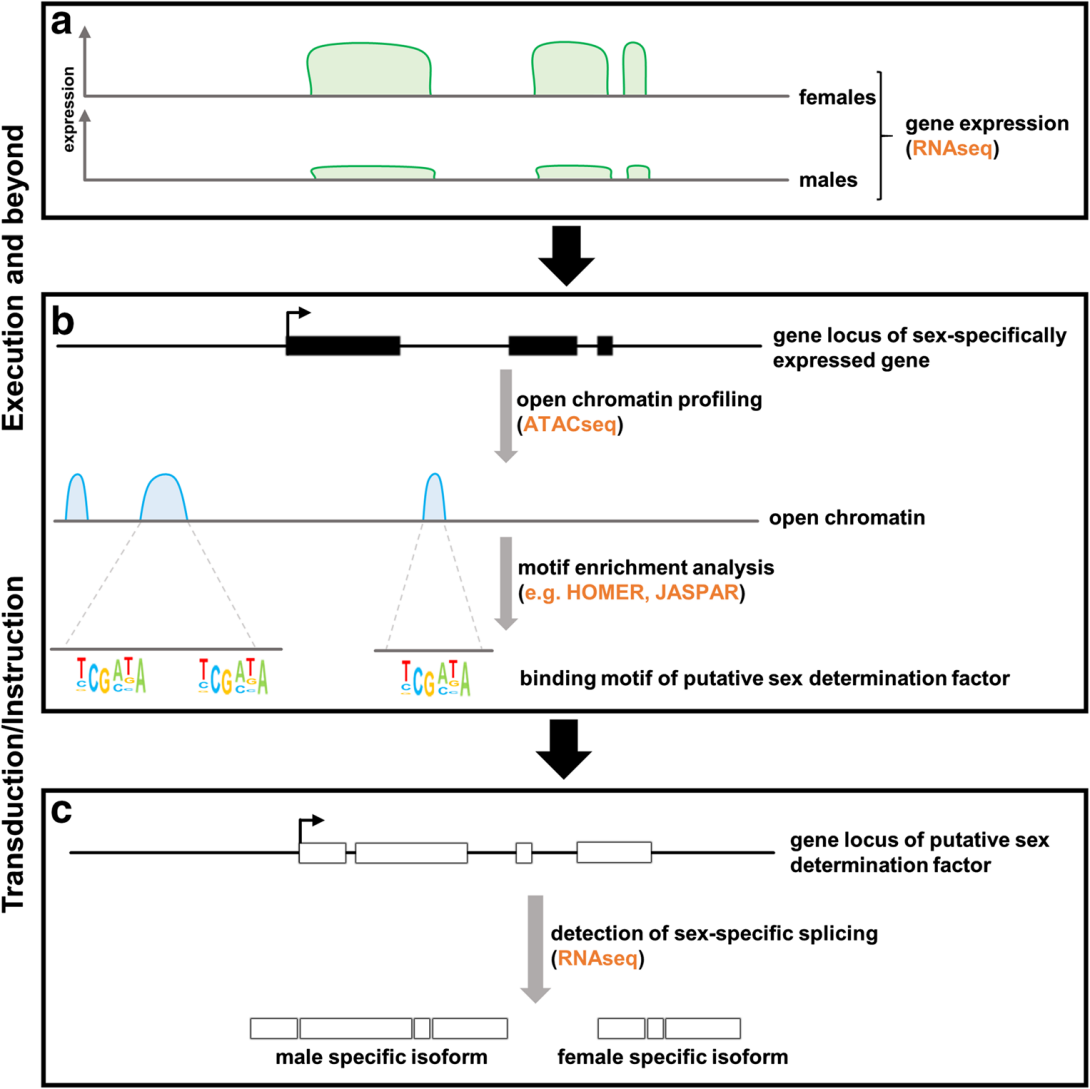
Overview of suggested unbiased methods to reveal sex-specifically expressed genes and factors involved in sex determination. (a) Schematic representation of quantitative RNAseq data showing higher expression of the gene in females compared with males. (b) Schematic representation of open chromatin profiling to reveal putative upstream regulators of sex-specifically expressed genes. (c) Isoform-specific RNAseq can be applied to identify sex-specific splice variants. Please refer to the main text for details and references

The final readout of embryonic sex determination is the sex-specific expression of genes, which control dimorphic organ development and later regulate dimorphic organ function. We broadly refer to this sex-specific readout as execution step. Therefore, one excellent method to identify genes regulating sex-specific organ development or function in spiders is studying sex-specific gene expression (Fig. 2a). It has for instance been shown that male and female spider toxins possess different activities and that the sexes produce different levels of toxins (Rash et al. 2000; de Oliveira et al. 1999). Intriguingly, a genome-wide survey of *latrotoxin* genes in the house spider *P. tepidariorum* revealed that a phylogenetically clustered group of these toxins is only expressed in males (Gendreau et al. 2017). Silk glands are as characteristic as venom glands for spiders, and due to their different dispersal and reproduction strategies (see above), female and male spiders have different requirements for the silk they produce. A genome-wide expression analysis of genes expressed in male and female silk glands in three Theridiidae species showed that indeed the complement of expressed genes is highly sex-specific. Interestingly, the authors found species-specific patterns of sex-specific silk gene expression, suggesting that males have different requirements in different species (Correa-Garhwal et al. 2017).

These examples demonstrate that the analysis of specific tissues is a powerful approach to identify organ-specifically expressed genes that show different expression profiles among sexes. While the findings exemplified above are based on the analysis of a few spider-specific organs, it is still unclear in how far other organs are characterized by sex-specific gene expression. Nowadays such genome-wide differential expression data between sexes can easily be obtained for a variety of species and tissues since next generation sequencing technologies facilitate the establishment of reference transcriptome resources (Grabherr et al. 2011; Haas et al. 2013). Since gene expression is highly dependent on the temporal and spatial context (reviewed in Buchberger et al. 2019), it is advisable to apply comparative gene expression studies to as specific tissue samples as possible. This way even low abundant transcripts can be detected in the tissue of interest. In summary, we argue that a comprehensive analysis of genome-wide expression differences in various organs and spider lineages will reveal common and lineage-specific aspects of sex-specific gene expression.

### Transduction

While the identification of sex-specific gene expression in adult spider organs provides insights into the functional organs, it remains to be established which molecular mechanisms control the sex-specific gene expression. Sex-specific gene expression on the execution level must be regulated beforehand by sex determining factors at the transduction level. Data on sex determination mechanisms in animals suggest that the number of key factors may be limited. Comparative studies showed that the transduction step involving differential splicing of a *tra* like gene and the subsequent activation of female- or male-specific Dsx transcription factors seems to be conserved in many insects (reviewed in Bopp et al. 2014). Indeed, *dsx* like genes that code for Doublesex and Male-abnormal-3 Related Transcription factors (Dmrt) have been implicated in sex-specific development in vertebrates (Matson et al. 2010; Yoshimoto et al. 2008), *C. elegans* (Mason et al. 2008; Shen and Hodgkin 1988), crustaceans (Kato et al. 2008; Kato et al. 2011), and planarians (Chong et al. 2013), making it a prime candidate to play a major role in specifying sex differences in spiders. A recent phylogenetic analysis of *Dmrt* genes in various arthropods showed that the *P. tepidariorum* genome contains one copy of the *Dmrt11E*, *Dmrt93B*, and *Dmrt99B* family, respectively, and two copies of *Dsx*-like genes (Panara et al. 2019). While *Dsx1* showed no expression during embryonic development, *Dsx2* (isoform B) expression has been observed in the embryonic anlagen of the spinnerets, which are highly sexually dimorphic structures in adult *P. tepidariorum* (Moon and An 2006; Panara et al. 2019)*.* Intriguingly, the same isoform of *Dsx2* has been shown to be differentially expressed in developing male and female pedipalps (Schomburg 2017), making *Dsx2* an excellent candidate for further functional studies (see below).

Although core events, such as the involvement of alternative splicing and *Dmrt* genes, are highly conserved in sex determination of arthropods studied to date, various lineage-specific aspects have also been revealed. For instance, in the silk moth *Bombyx mori* the cascade leading to female-specific *Dsx* splicing does not rely on the presence of a *tra* gene but is initiated by the expression of a PIWI-interacting RNA (piRNA) (Kiuchi et al. 2014). In the light of the diversity in aspects of the sex determination cascade, it may be risky to rely on the usual suspects only in the search for candidate genes in spiders. To identify sex determining factors independently from a candidate gene approach, unbiased genome-wide approaches are necessary. If the number of genomic loci contributing to the core of the sex determination cascade is limited, one could identify common upstream regulators of genes with sex-specific expression. To this end, the regulatory sequences of genes of interest could be surveyed for transcription factor binding motifs using established tools such as HOMER (Heinz et al. 2010). Since motif information specifically for spiders is sparse, one could query existing motif databases, such as JASPAR (Mathelier et al. 2016; Sandelin et al. 2004) to link identified motifs to putative transcription factors (Fig. 2b). User friendly tools to statistically evaluate the putative enrichment of motifs in a set of sex specifically expressed genes have been established (e.g., Herrmann et al. 2012). However, a key prerequisite for such a proposed analysis is the availability of high-quality genomes with comprehensive gene model annotations that allow identifying potential regulatory sequences. An increasing number of spider genomes are being sequenced these days. Since spider genomes tend to be rather large (for a recent overview see Garb et al. 2018), it may be additionally important to narrow down relevant regulatory sequences. This could be achieved by generating stage and tissue specific ATAC-seq datasets to assess genome-wide chromatin accessibility (Buenrostro et al. 2013, 2015) (Fig. 2b).

Besides the involvement of *Dmrt* genes in sex determination, differential splicing seems to be a common theme in many insects studied so far (Salz 2011). As outlined above, *D. melanogaster* sex determination involves male- and female-specific splicing of *tra* that itself codes for a splicing factor. Eventually, sex-specific *Dsx* isoforms activate male- and female-specific gene expression. Recent work revealed that the male determining factor in the house fly *M. domestica* is a paralog of a general splicing factor (Sharma et al. 2017). Although differential splicing does not seem to play a major role in *C. elegans* sex determination (Cline and Meyer 1996), it has been suggested that the male determinant *sex-determining region on Y* (*Sry*) in mammals codes for a transcriptional regulator that is also involved in pre-mRNA splicing (Lalli et al. 2003). Since sex-specific splicing is involved in many organisms, one approach to identify genes involved in spider sex determination could be to identify such events in different tissues and developmental stages (Fig. 2c). Several methods have been established to infer alternative splicing events from RNA sequencing data. Many pipelines require the a well-annotated genome reference, but *de novo* transcriptome assemblies can also be used to identify and quantify differential splicing events (Benoit-Pilven et al. 2018). The availability of an increasing number of transcriptomic datasets in spiders will therefore allow to re-analyze this data with respect to sex-specific splicing events. Hence, the application of genomic and transcriptomic resources and comparative tools will contribute to a better understanding of sex determination mechanisms in spiders.

### Instruction

In contrast to transduction, instruction is very variable in all arthropod species studied so far. A few mechanisms identified in animals comprise the above described autosome to gonosome ratio (*Drosophila*), male determining factors that can be located on any chromosome (*Musca*), signals coming from the presence of a Y chromosome or its absence (*Aedes aegypti*), and ploidy level (Hymenoptera) (but see Herpin and Schartl 2015 for a review). Sequences homologous to *sex lethal* and *csd* can be found in spider genomes available to date. However, they do not necessarily have the same function and warrant further inspection. Lack of conservation in the instruction upstream of *tra* makes a candidate approach based on orthology unlikely to be successful, more so because phylogenetic distance between insects and spiders is considerable.

To date, the karyotypes of 867 species of spiders were characterized through cytogenetic analyses, and a comprehensive review and database were compiled by Araujo et al. (2012), www.arthropodacytogenetics.bio.br/spiderdatabase. This compilation of studies, some of them published at the turn of the twentieth century, not only contributes to our understanding of karyotype evolution in spiders but also delivers insights into the sex chromosome system of many of them. Thanks to this substantial body of literature, one can hypothesize which of the induction mechanisms listed above might act.

A variation of the male heterogametic XY sex determination system, X_1_X_2_0, is found most frequently throughout all spider genera. In this system, female karyotypes are 2n=autosomes+ X_1_X_1_X_2_X_2_ and male karyotypes are 2n=autosomes+ X_1_X_2_0. The X_1_X_2_0 SCS is considered a plesiomorphic feature in spiders because it occurs in members of the phylogenetically basal family Liphistiidae (Mesothelae) (Suzuki 1954). In most of the spider taxa karyotyped so far, we can thus exclude a role of the Y chromosome. Further, a mechanism found in haplodiploid species such as some Hymenopterans, where the ploidy level determines offspring sex, is highly improbable. Instruction through either X:A ratios or X chromosome number would thus be the first working hypothesis in spiders.

### Functional validation of candidate genes

The function of candidate genes identified by genome-wide approaches could be tested by gene knockdown during embryonic development applying RNA interference (RNAi). This method is well-established in *P. tepidariorum* where RNAi is systemic and parental, meaning that the injection of double-stranded RNA in mothers results in efficient gene knockdown in their offspring (Hilbrant et al. 2012). Subsequently, offspring at the subadult instar stages and older should be investigated with respect to their pedipalp morphology and the formation of the epigyne to determine the phenotypic sex. An informative readout of the experiment could be the analysis of the sex ratio among offspring within cocoons that originate from injected and control females, respectively. If the adult sex ratio is significantly different in RNAi-treated cocoons, the targeted gene most likely interferes with the sex determination pathway. Such an approach could be significantly improved if the phenotypic sex could be determined much earlier during development. One reason for the need of an early sex identification is the observation that the RNAi effect ceases over time and gene expression often recovers in later stages of development. Additionally, one could significantly reduce the time and resource investment if the spiders would not have to be kept until adulthood. Therefore, more comparative morphological studies are needed to identify external and internal features that show differences between sexes. Additionally, a proper molecular characterization of the genotypic sex will facilitate the establishment of efficient assays to determine the sex for each individual in RNAi and control experiments. A discrepancy between the genetic and the phenotypic sex could be used to identify sex determining genes in RNAi experiments.

In summary, extensive knowledge acquired in a few model systems provides a solid basis to start searching for genes involved in sex determination in spiders following a candidate gene approach. The increasing number of established genomic resources furthermore facilitates the unbiased identification of putative lineage-specific sex determining factors. Eventually, identified candidate genes can be functionally tested using RNAi-mediated gene knockdown. These exciting developments in sequencing technology and functional assays in combination with the extensive sexual dimorphism in various phenotypic traits such as morphology, behavior, and life history make spiders an excellent model to study the genetic basis, the phenotypic consequences, and the evolutionary forces underlying sexual dimorphism.

### Sex chromosome evolution: what can we learn from spiders

As aforementioned, the instruction of sex differentiation is a highly variable mechanism in animals, and even varies substantially between members of the same genus or the same species, in extreme cases. Indeed, Hamm et al. (2015) described how the male determining factor is localized on different chromosome in different *Musca* populations. In species with genetic sex determination, such as spiders, sex chromosomes are thought to be key to the establishment of separate male and female phenotype. Vicoso (2019) very recently reviewed data on the variability of sex-determining genes and sex chromosomes in non-model species and more especially invertebrates. This review highlights the incredible diversity of sex-determining systems, and shows the importance of broadening our knowledge by sampling all biological diversity to better understand sex chromosome evolution.

Various hypotheses concerning the origin of the X_1_X_2_0 SCS system in spiders have been put forth and are reviewed in Araujo et al. (2012). Competing hypotheses include but are not limited to (1) duplication of the ancestral single X (Revell 1947) or (2) centric fission of this ancestral single X and additional rearrangements (Pätau 1948). In both cases, these events would have been followed by differentiation and enhanced by a lack of recombination between the two X, leading to them becoming X_1_ and X_2_. All authors agree on the partial or complete lack of homology between these X chromosomes (Hackman 1948, Suzuki 1952, Mittal 1964). Král et al. (2006) revealed that while the X_1_X_2_0 sex chromosome system predominates in entelegyne spiders, it has been found in only two genera of basal araneomorphs. Systems with 3 X chromosomes or more, as well as SCS with Y chromosomes, are thought to be derived from the ancestral karyotype (see e.g. Datta and Chatterjee 1988 for Araneids and references in Araujo et al. 2012). Some genera are highly diverse in this regard, with numerous neo Y chromosomes appearing in *Habronattus* spiders, for example (Maddison and Leduc-Robert 2013).

To date, all analyses and hypotheses about the evolution of X chromosomes in spiders rely on classical cytogenetic methods, measuring basic chromosomal characteristics such as chromosome length, number, meiotic condensation, meiotic segregation, and cell ultrastructure (e.g. Král et al. 2006 and citations in Araujo et al. 2012). Little has been done in the way of gene content on X chromosomes or X-linked regions. This is not surprising given the relatively new availability of whole genome sequencing techniques. In terms of genome sequencing, spiders are lagging behind other groups (reviewed in Garb et al. 2018), partly because of their sheer size (between 723 Mb and 5.60 Gb, Gregory and Shorthouse 2003). However, various affordable approaches are suitable for X-linked region discovery and ultimately analysis of X chromosome evolution. The difficulty to obtain sex chromosome sequences is increasingly being overcome by methodological advances (reviewed in Muyle et al. 2017; Palmer et al. 2019). Promising methods associate reasonable sequencing volume and high sensitivity, such as SEX-DETector (Muyle et al. 2016). One of its advantages is its reliance on RNAseq data, as opposed to DNAseq. The procedure analyzes allelic segregation in a family with two parents and around 5 offspring of each sex obtained through a controlled cross. This allows circumventing issues with genome assembly for species with large genomes and is also more cost effective. Other methods include the sequencing of one individual of each sex and depth of coverage distribution. This method relies on the assumption that any X-linked genome region would be twice more abundant in females than in males. Such differential coverage analyses have been successfully applied to snakes (Vicoso et al. 2013) and Lepidoptera (Fraisse et al. 2017), among others. Recent work aiming at finding sex-linked regions in the tarantula *Grammostola rosea* led to the identification of 16 X-chromosome-linked molecular markers for use in a quantitative PCR approach (pers. communication Petr Nguyen, MSc. thesis Pechová (2018)). Among these, two were also found to be sex linked in *P. tepidariorum*, despite the two species being phylogenetically very distant.

The sex-specific X chromosome count has driven its specialization, and understanding the forces that drive this specialization has been a longstanding goal of evolutionary biology (Charlesworth et al. 2005). X chromosomes are under a peculiar selection regime because they are transmitted through females two-third of the times; this may lead to the acquisition of specialized gene content. X chromosomes will accumulate an excess of dominant mutations that are beneficial to females (Rice 1984), potentially leading to the accumulation of genes with female functions on this chromosome. The exposure of recessive alleles to selection in males causes higher substitution rates in X-linked loci, a phenomenon termed faster X hypothesis. The evolution of the X chromosome also regards its gene content. While high gene turnover on X chromosomes has been observed in Dipteran insects (Vicoso and Bachtrog 2015), a study on Hemiptera showed that differentiated sex chromosomes are extremely stable (Pal and Vicoso 2015). Bechsgaard et al. (2019) very recently published the first study on the evolution of X-linked regions in spiders. Their approach made clever use of flow cytometry to sort sperm cells from the social spider *Stegodyphus mimosarum* into two groups, those containing X chromosomes and those who did not. Reduced representation libraries were then sequenced, and X chromosome-to-autosome diversity estimated. This study provides evidence in support of the faster X hypothesis in spiders and calls for further investigation in other spiders.

Further, the X often evolves mechanisms of dosage compensation, which regulate the expression of X-linked genes to compensate for their haploidy in males (Charlesworth 1998). Sex chromosome dosage compensation (SCDC) is achieved in different ways depending on the species, and regarded as complete or partial, depending on whether all X-linked genes are affected or only a fraction, respectively. While in mammals a whole X chromosome copy is inactivated in genetic females (Heard et al. 1997), in *D. melanogaster* the expression of X-linked genes is upregulated in males (reviewed in Lucchesi and Kuroda 2015). This mode of dosage compensation is broadly conserved across flies (Vicoso and Bachtrog 2015) and seems also predominant in other insects (e.g., stalk-eyed fly, Australian sheep blowfly and other references in Gu and Walters 2017). Continuing to broaden the scope of taxa in which dosage compensation has been assayed is one clear path forward to further our understanding of sex chromosome evolution and SCDC (Gu & Walters 2017).

## Open questions and future challenges

### Sexing at all developmental stages

Until now, sex could not be determined until later stages of development without rendering further analysis impossible: chromosome spreads are destructive, as is sperm sorting through flow cytometry. To understand the cascade of sex determining events and the genes involved in instruction and transduction in spiders, a holistic approach must be applied. Reliable methods must be employed to either manipulate offspring sex or to determine the sex at any stage. The former could be achieved through artificial insemination with sperm sorted through flow cytometry and thus leading to female or male-only broods. The latter would be possible once molecular markers for X chromosome are available, and their relative copy number to autosome-linked regions can be quantified through quantitative PCR (D'Haene et al. 2010; Hoebeeck et al. 2007; for an example in reptiles see Rovatsos and Kratochvíl 2017). If X chromosomes are conserved throughout Araneids, one could easily use the knowledge gained on one species to apply the molecular markers to another species.

### Monogenic vs. polygenic sex determination

So far it is unclear whether single loci on sex chromosomes contribute to sex determination, as is common in *D. melanogaster*, *C. elegans*, and most mammals, or if this process is controlled by multiple genes, such as in zebrafish (Bradley et al. 2011). Interestingly, it has been proposed that the involvement of multiple genes may be an indication for a more recent split of females and males from a hermaphroditic ancestor. In this case, at least two genes are necessary to define the two sexes, one factor that suppresses the female fate and one that suppresses the male fate. In contrast, the presence of one central sex determining factor may be the result of long-term establishment of such a system (Bachtrog et al. 2014). Therefore, the identification of the number of sex determining genes in spiders may have a direct implication for the evolution of sexes.

### Nonautonomous vs. cell-autonomous sex determination

Dimorphic development can be coordinated by two main mechanisms. If the sex determining signal is interpreted in each cell from early stages on, sex-specific gene expression and thus development is triggered cell-autonomously. This is the typical mode observed in *D. melanogaster* (Cline 1993). In mammals, however, dimorphic development is triggered by nonautonomous signals, such as hormones which are mostly secreted from the gonads (Wilhelm et al. 2007). It may be interesting to find out whether sex determination in spiders is coordinated cell-autonomously or non-autonomously. Once a thorough anatomical description of sexually dimorphic development will be available, one could compare the earliest stages that show morphological differences between sexes for different organs. If dimorphic development is highly coordinated in various organs, it is likely that cell-autonomous processes are at play from early stages on. In contrast, the dimorphic development of different organs at different stages may imply that inductive nonautonomous processes are at play.

### Sexual dimorphism in growth plasticity

As outlined above, growth in spiders is highly plastic and males and females respond differently to external cues. It is generally accepted that plasticity in response to the environment is often associated with epigenetic modifications, such as DNA methylation and histone modifications (Cavalli 2006; Duncan et al. 2014; Fagiolini et al. 2009). It will thus be of major interest to unravel the general contribution of epigenetic modifications to phenotypic plasticity in spiders. First results of genome-wide bisulfite sequencing to study methylation patterns in the social spider *Stegodyphus dumicola* showed a higher level of methylation in gene loci and a link between methylation and gene expression has been established. A comparative genomics analysis included in this study furthermore suggests that DNA methylation is common in spiders (Liu et al. 2019). With a general understanding of the epigenetic and genetic mechanisms underlying phenotypic plasticity, it will be exciting to test whether epigenetic factors are somehow linked to the sex determination cascade in spiders to drive sex-specific responses to an ever-changing environment. With the availability of more high-quality genomes in the future, it will be possible to comprehensively study the epigenetic landscape in spiders with respect to sexual dimorphisms in plasticity on a macroevolutionary as well as microevolutionary scale.

### Evolution of sex ratios

While many animals possess a 1:1 sex ratio, skewed sex ratios are similarly pervasive. In some social insects the sex is determined by the haplodiploidy system in which unfertilized haploid eggs give rise to males, while fertilized diploid eggs develop into females (Gardner and Ross 2013). With this system, the sex ratio can be directly controlled. Another source of skewed sex ratio is the unequal transmission of X or Y chromosomes, a process known as sex chromosome meiotic drive. Meiotic drives can be caused by mutations in sex chromosomes that interfere with meiosis, or they can be induced by endosymbionts to ensure their propagation (Hurst and Jiggins 2000; Jaenike 2001). Interestingly, social spiders have skewed sex ratios towards females (Vollrath 1986), while it is more equal in solitary spiders. Since spiders have diploid chromosomal sex determination the ratio cannot be controlled directly as in a haplodiploid system, where unfertilized eggs develop into males. A recent study in two social spider species with female-biased sex ratio (*Stegodyphus dumicola* and *S. mimosarum*) and one subsocial species with a 1:1 sex ratio (*S. africanus*) showed that the bias in social spiders is achieved on the level of sperm production. The analysis of the DNA content of the sperm showed that much more sperm cells with X chromosomes were produced which leads to an excess of females. These findings are consistent with X chromosome meiotic drive. Furthermore, the authors provide data suggesting that endosymbionts do not cause the drive (Vanthournout et al. 2018). In addition to sperm analysis, sex-specific markers could be used on spiderlings at different developmental stages to assess whether male killing or any other sex-biased mortality occurs in broods. It will be highly interesting to reveal the mechanisms driving skewed sex ratios in different spider lineages to contribute to a better understanding of the genomic basis of this process.

## Conclusion

Once key mechanisms underlying sex determination in spiders are unraveled, we will able to revisit many interesting phenomena in spiders, ranging from sex allocation, sex-specific growth strategies, adaptive variation in early sex-differences in developmental pathways to sex differences in dispersal - to name just a short selection of the topics. The findings obtained in spiders will contribute to a better general understanding of the evolution of sexual dimorphism and the underlying mechanisms. They will further elucidate the developmental responses to biotic and abiotic factors that can cause plasticity in physiology, morphology, and behavior.

## References

[CR1] Agrawal AF (2001). Sexual selection and the maintenance of sexual reproduction. Nature..

[CR2] Aisenberg A, González M (2011). Male mate choice in *Allocosa alticeps* (Araneae: Lycosidae), a sand-dwelling spider with sex role reversal. J Arachnol.

[CR3] Aisenberg A, Viera C, Costa FG (2007). Daring females, devoted males, and reversed sexual size dimorphism in the sand-dwelling spider *Allocosa brasiliensis* (Araneae, Lycosidae). Behav Ecol Sociobiol.

[CR4] Akiyama-Oda Y, Oda H (2006). Axis specification in the spider embryo: dpp is required for radial-to-axial symmetry transformation and sog for ventral patterning. Development.

[CR5] Anderson JT, Morse DH (2001). Pick-up lines: cues used by male crab spiders to find reproductive females. Behav Ecol.

[CR6] Andersson MB (1994) Sexual selection. Princeton University Press

[CR7] Andrade MCB (1996). Sexual selection for male sacrifice in the Australian redback spider. Science..

[CR8] Andrade MCB (2019) Sexual selection and social context: web-building spiders as emerging models for adaptive plasticity. In: Advances in the study of behavior, vol 51. Elsevier, pp 177–250. 10.1016/bs.asb.2019.02.002

[CR9] Andrade MCB, Gu L, Stoltz JA (2005). Novel male trait prolongs survival in suicidal mating. Biol Lett.

[CR10] Araujo D, Schneider MC, Paula-Neto E, Cella DM, Swan A (2012). Sex chromosomes and meiosis in spiders: a review. Meiosis: molecular mechanisms and cytogenetic diversity.

[CR11] Arnqvist G, Rowe L (2005) Sexual conflict. Princeton University Press

[CR12] Aviles L, Purcell J (2012) The evolution of inbred social systems in spiders and other organisms: from short-term gains to long-term evolutionary dead ends? In: Brockmann HJ, Roper TJ, Naguib M, Mitani JC, Simmons LW (eds) Advances in the study of behavior, vol 44. Advances in the Study of Behavior, pp 99–133. 10.1016/b978-0-12-394288-3.00003-4

[CR13] Bachtrog D et al (2014) Sex determination: why so many ways of doing it? PLoS Biol 12. 10.1371/journal.pbio.1001899 10.1371/journal.pbio.1001899 PMC4077654 24983465

[CR14] Barth FG (2002). Spider senses - technical perfection and biology. Zoology..

[CR15] Bateman AJ (1948). Intra-sexual selection in *Drosophila*. Heredity..

[CR16] Bechsgaard J, Schou MF, Vanthournout B, Hendrickx F, Knudsen B, Settepani V, Schierup MH, Bilde T (2019). Evidence for faster X chromosome evolution in spiders. Mol Biol Evol.

[CR17] Becker E, Riechert S, Singer F (2005). Male induction of female quiescence/catalepsis during courtship in the spider, *Agelenopsis aperta*. Behaviour..

[CR18] Bell G (1982). The masterpiece of nature: the evolution and genetics of sexuality.

[CR19] Benoit-Pilven C, Marchet C, Chautard E, Lima L, Lambert MP, Sacomoto G, Rey A, Cologne A, Terrone S, Dulaurier L, Claude JB, Bourgeois CF, Auboeuf D, Lacroix V (2018). Complementarity of assembly-first and mapping-first approaches for alternative splicing annotation and differential analysis from RNAseq data. Sci Rep.

[CR20] Beyer M, Czaczkes TJ, Tuni C (2018). Does silk mediate chemical communication between the sexes in a nuptial feeding spider?. Behav Ecol Sociobiol.

[CR21] Biaggio MD, Sandomirsky I, Lubin Y, Harari AR, Andrade MCB (2016) Copulation with immature females increases male fitness in cannibalistic widow spiders. Biol Lett 12. 10.1098/rsbl.2016.0516 10.1098/rsbl.2016.0516 PMC5046930 27651535

[CR22] Blanckenhorn WU (2005). Behavioral causes and consequences of sexual size dimorphism. Ethology..

[CR23] Bonte D, Hovestadt T, Poethke HJ (2009) Sex-specific dispersal and evolutionary rescue in metapopulations infected by male killing endosymbionts. BMC Evol Biol:9. 10.1186/1471-2148-9-16 10.1186/1471-2148-9-16 PMC2633281 19149895

[CR24] Bopp D, Saccone G, Beye M (2014). Sex determination in insects: variations on a common theme. Sex Dev.

[CR25] Bradley KM, Breyer JP, Melville DB, Broman KW, Knapik EW, Smith JR (2011). An SNP-based linkage map for zebrafish reveals sex determination loci. G3: genes, genomes. Genetics..

[CR26] Buchberger E, Reis M, Lu T-H, Posnien N (2019) Cloudy with a chance of insights: context dependent gene regulation and implications for evolutionary studies. Genes. 10. 10.3390/genes10070492 10.3390/genes10070492 PMC6678813 31261769

[CR27] Buenrostro JD, Giresi PG, Zaba LC, Chang HY, Greenleaf WJ (2013). Transposition of native chromatin for fast and sensitive epigenomic profiling of open chromatin, DNA-binding proteins and nucleosome position. Nat Methods.

[CR28] Buenrostro JD, Wu B, Chang HY, Greenleaf WJ (2015). ATAC-seq: a method for assaying chromatin accessibility genome-wide. Curr Prot Mol Biol.

[CR29] Bulmer MG, Parker GA (2002). The evolution of anisogamy: a game-theoretic approach. Proc R Soc B Biol Sci.

[CR30] Cavalli G (2006). Chromatin and epigenetics in development: blending cellular memory with cell fate plasticity. Development..

[CR184] Charlesworth B (1998) Sex chromosomes: Evolving dosage compensation. Current Biology 8 (25):R931–R933. 10.1016/S0960-9822(98)00013-X 10.1016/s0960-9822(98)00013-x 9889089

[CR31] Charlesworth D, Willis JH (2009). The genetics of inbreeding depression. Nat Rev Genet.

[CR32] Charlesworth D, Charlesworth B, Marais G (2005). Steps in the evolution of heteromorphic sex chromosomes. Heredity..

[CR33] Chiara V, Ramon Portugal F, Jeanson R (2019) Social intolerance is a consequence, not a cause, of dispersal in spiders. PLoS Biol 17. 10.1371/journal.pbio.3000319 10.1371/journal.pbio.3000319 PMC6605646 31265448

[CR34] Cho M, Neubauer P, Fahrenson C, Rechenberg I (2018) An observational study of ballooning in large spiders: nanoscale multifibers enable large spiders’ soaring flight. PLoS Biol 16. 10.1371/journal.pbio.2004405 10.1371/journal.pbio.2004405 PMC6001951 29902191

[CR35] Chong T, Collins JJ, Brubacher JL, Zarkower D, Newmark PA (2013). A sex-specific transcription factor controls male identity in a simultaneous hermaphrodite. Nat Commun.

[CR36] Cline TW (1993). The *Drosophila* sex determination signal: how do flies count to two?. Trends Genet.

[CR37] Cline TW, Meyer BJ (1996). Vive la différence: males vs females in flies vs worms. Annu Rev Genet.

[CR38] Correa-Garhwal SM, Chaw RC, Clarke TH, Ayoub NA, Hayashi CY (2017). Silk gene expression of theridiid spiders: implications for male-specific silk use. Zoology..

[CR39] Datta SN, Chatterjee K (1988). Chromosomes and sex determination in 13 araneid spiders of north-eastern India. Genetica..

[CR183] de Oliveira KC, Gonçalves de Andrade RM, Giusti AL, Dias da Silva W, Tambourgi DV (1999) Sex-linked variation of *Loxosceles intermedia* spider venoms. Toxicon 37 (1):217–221. 10.1016/s0041-0101(98)00130-5 10.1016/s0041-0101(98)00130-5 9920493

[CR40] D'Haene B, Vandesompele J, Hellemans J (2010). Accurate and objective copy number profiling using real-time quantitative PCR. Methods..

[CR41] Duncan EJ, Gluckman PD, Dearden PK (2014). Epigenetics, plasticity, and evolution: how do we link epigenetic change to phenotype?. J Exp Zool B Mol Dev Evol.

[CR42] Eberhard WG (1980). The natural history and behavior of the bolas spider *Mastophora dizzydeani* sp. n.(Araneidae). Psyche J Entomol.

[CR43] Elgar MA, Schneider JM (2004). The evolutionary significance of sexual cannibalism. Advances in the Study of Behavior.

[CR44] Erez T, Schneider JM, Lubin Y (2005). Is male cohabitation costly for females of the spider *Stegodyphus lineatus* (Eresidae)?. Ethology..

[CR45] Fagiolini M, Jensen CL, Champagne FA (2009). Epigenetic influences on brain development and plasticity. Curr Opin Neurobiol.

[CR46] Fairbairn DJ (2013) Odd couples: extraordinary differences between the sexes in the animal kingdom. Princeton University Press

[CR47] Fernández-Montraveta C, Marugán-Lobón J (2017). Geometric morphometrics reveals sex-differential shape allometry in a spider. Peerj..

[CR48] Fischer A (2019). Chemical communication in spiders - a methodological review. J Arachnol.

[CR49] Foelix RF (2011). Biology of spiders.

[CR50] Foellmer MW, Moya-Larano J (2007) Sexual size dimorphism in spiders: patterns and processes. In: Sex, size and gender roles: Evolutionary studies of sexual size dimorphism. Oxford University Press, Oxford, pp 71-81

[CR51] Fraisse C, Picard MAL, Vicoso B (2017). The deep conservation of the Lepidoptera Z chromosome suggests a non-canonical origin of the W. Nat Commun.

[CR52] Fromhage L, Schneider JM (2005). Safer sex with feeding females: sexual conflict in a cannibalistic spider. Behav Ecol.

[CR53] Fromhage L, Schneider JM (2012). A mate to die for? A model of conditional monogyny in cannibalistic spiders. Ecol Evol.

[CR54] Gamble T, Zarkower D (2012). Sex determination. Curr Biol.

[CR55] Garb JE, Sharma PP, Ayoub NA (2018). Recent progress and prospects for advancing arachnid genomics. Curr Opin Insect Sci.

[CR56] Gardner A, Ross L (2013). Haplodiploidy, sex-ratio adjustment, and eusociality. Am Nat.

[CR57] Gendreau KL, Haney RA, Schwager EE, Wierschin T, Stanke M, Richards S, Garb JE (2017). House spider genome uncovers evolutionary shifts in the diversity and expression of black widow venom proteins associated with extreme toxicity. BMC Genomics.

[CR58] Grabherr MG, Haas BJ, Yassour M, Levin JZ, Thompson DA, Amit I, Adiconis X, Fan L, Raychowdhury R, Zeng Q, Chen Z, Mauceli E, Hacohen N, Gnirke A, Rhind N, di Palma F, Birren BW, Nusbaum C, Lindblad-Toh K, Friedman N, Regev A (2011). Full-length transcriptome assembly from RNA-Seq data without a reference genome. Nat Biotechnol.

[CR59] Gregory T, Shorthouse D (2003). Genome sizes of spiders. J Hered.

[CR60] Gu LQ, Walters JR (2017). Evolution of sex chromosome dosage compensation in animals: a beautiful theory, undermined by facts and bedeviled by details. Genome Biol Evol.

[CR61] Haas BJ, Papanicolaou A, Yassour M, Grabherr M, Blood PD, Bowden J, Couger MB, Eccles D, Li B, Lieber M, MacManes M, Ott M, Orvis J, Pochet N, Strozzi F, Weeks N, Westerman R, William T, Dewey CN, Henschel R, LeDuc R, Friedman N, Regev A (2013). De novo transcript sequence reconstruction from RNA-seq using the trinity platform for reference generation and analysis. Nat Protoc.

[CR186] Hackman W (1948) Chromosomenstudien an Araneen mit besonderer berücksichtigung der Geschlechtschromosomen. Acta Zoologica Fennica 54:1-101

[CR62] Hamm RL, Meisel RP, Scott JG (2015) The evolving puzzle of autosomal versus Y-linked male determination in *Musca domestica*. G3: genes, genomes. Genetics. 5:371–384. 10.1534/g3.114.014795 10.1534/g3.114.014795 PMC4349091 25552607

[CR63] Head G (1995). Selection on fecundity and variation in the degree of sexual size dimorphism among spider species (Class Araneae). Evolution..

[CR64] Heard E, Clerc P, Avner P (1997). X-chromosome inactivation in mammals. Annu Rev Genet.

[CR65] Hebets EA, Papaj DR (2005). Complex signal function: developing a framework of testable hypotheses. Behav Ecol Sociobiol.

[CR66] Heinz S, Benner C, Spann N, Bertolino E, Lin YC, Laslo P, Cheng JX, Murre C, Singh H, Glass CK (2010). Simple combinations of lineage-determining transcription factors prime cis-regulatory elements required for macrophage and B cell identities. Mol Cell.

[CR67] Herberstein M, Schneider J, Uhl G, Michalik P (2011). Sperm dynamics in spiders. Behav Ecol.

[CR68] Herberstein ME, Wignall AE, Hebets EA, Schneider JM (2014). Dangerous mating systems: signal complexity, signal content and neural capacity in spiders. Neurosci Biobehav Rev.

[CR69] Herpin A, Schartl M (2015). Plasticity of gene-regulatory networks controlling sex determination: of masters, slaves, usual suspects, newcomers, and usurpators. EMBO Rep.

[CR70] Herrmann C, van de Sande B, Potier D, Aerts S (2012). i-cisTarget: an integrative genomics method for the prediction of regulatory features and cis-regulatory modules. Nucleic Acids Res.

[CR71] Hilbrant M, Damen WGM, McGregor AP (2012). Evolutionary crossroads in developmental biology: the spider *Parasteatoda tepidariorum*. Development..

[CR72] Hoebeeck J, Speleman F, Vandesompele J (2007) Real-time quantitative PCR as an alternative to southern blot or fluorescence in situ hybridization for detection of gene copy number changes. In: Hilario E, Mackay J (eds) Protocols for nucleic acid analysis by nonradioactive probes, vol 353. Methods in Molecular Biology. Springer, pp 205–226 10.1385/1-59745-229-7:205 17332643

[CR73] Hormiga G (2000) Higher level phylogenetics of erigonine spiders (Araneae, Linyphiidae, Erigoninae). Smithsonian Contributions to Zoology

[CR74] Huber BA (1995). Copulatory mechanism in *Holocnemus pluchei* and *Pholcus opilionoides*, with notes on male cheliceral apophyses and stridulatory organs in Pholcidae (Araneae). Acta Zool.

[CR75] Huber BA (2005). Sexual selection research on spiders: progress and biases. Biol Rev.

[CR76] Huber BA, Nuñeza OM (2015). Evolution of genital asymmetry, exaggerated eye stalks, and extreme palpal elongation in Panjange spiders (Araneae: Pholcidae). Eur J Taxon.

[CR77] Huber BA, Pérez-González A, Astrin JJ, Blume C, Baptista R (2013). *Litoporus iguassuensis* (Araneae, Pholcidae): camouflaged retreat, sexual dimorphism, female color polymorphism, intra-specific genital variation, and description of the male. Zoologischer Anzeiger – J Comp Zool.

[CR78] Hurst GD, Jiggins FM (2000). Male-killing bacteria in insects: mechanisms, incidence, and implications. Emerg Infect Dis.

[CR79] Hussin JG, Hodgkinson A, Idaghdour Y, Grenier JC, Goulet JP, Gbeha E, Hip-Ki E, Awadalla P (2015). Recombination affects accumulation of damaging and disease-associated mutations in human populations. Nat Genet.

[CR80] Jaenike J (2001). Sex chromosome meiotic drive. Annu Rev Ecol Syst.

[CR81] Janzen FJ, Paukstis GL (1991). Environmental sex determination in reptiles: ecology, evolution, and experimental design. Q Rev Biol.

[CR82] Johnson JC, Halpin R, Stevens D, Vannan A, Lam J, Bratsch K (2015). Individual variation in ballooning dispersal by black widow spiderlings: the effects of family and social rearing. Curr Zool.

[CR83] Johnson JC, Halpin R, Stevens DR (2016). Extreme developmental synchrony reduces sibling cannibalism in the black widow spider, *Latrodectus hesperus*. Anim Behav.

[CR84] Kato Y, Kobayashi K, Oda S, Colbourn JK, Tatarazako N, Watanabe H, Iguchi T (2008). Molecular cloning and sexually dimorphic expression of DM-domain genes in *Daphnia magna*. Genomics..

[CR85] Kato Y, Kobayashi K, Watanabe H, Iguchi T (2011). Environmental sex determination in the branchiopod crustacean *Daphnia magna*: deep conservation of a *Doublesex* gene in the sex-determining pathway. PloS Genet.

[CR86] Kiuchi T, Koga H, Kawamoto M, Shoji K, Sakai H, Arai Y, Ishihara G, Kawaoka S, Sugano S, Shimada T, Suzuki Y, Suzuki MG, Katsuma S (2014). A single female-specific piRNA is the primary determiner of sex in the silkworm. Nature..

[CR87] Kleinteich A, Schneider J (2010). Evidence for Rensch’s rule in an orb-web spider with moderate sexual size dimorphism. Evol Ecol Res.

[CR88] Kotiaho J, Alatalo R, Mappes J, Parri S (1996). Sexual selection in a wolf spider - male drumming activity, body-size, and viability. Evolution..

[CR89] Kovoor J, Peters HM (1988). The spinning apparatus of *Polenecia producta* (Araneae, Uloboridae): structure and histochemistry. Zoomorphology..

[CR188] Král JJ, Musilová J, Št’áhlavský J, Řezáč M, Akan Z, Edwards RL, Coyle FA, Ribera Almerje C (2006) Evolution of the karyotype and sex chromosome systems in basal clades of araneomorph spiders (Araneae: Araneomorphae). Chromosome Research 14: 859. https://doi.org/10.1007/s10577-006-1095-9 10.1007/s10577-006-1095-9 17195053

[CR90] Kuntner M, Coddington JA (2020). Sexual size dimorphism: evolution and perils of extreme phenotypes in spiders. Annu Rev Entomol.

[CR91] Kuntner M, Zhang SC, Gregoric M, Li DQ (2012). *Nephila* female gigantism attained through post-maturity molting. J Arachnol.

[CR92] Lalli E, Ohe K, Latorre E, Bianchi ME, Sassone-Corsi P (2003). Sexy splicing: regulatory interplays governing sex determination from *Drosophila* to mammals. J Cell Sci.

[CR93] Li XY, Kokko H (2019). Sex-biased dispersal: a review of the theory. Biol Rev.

[CR94] Ligon RA et al (2018) Evolution of correlated complexity in the radically different courtship signals of birds-of-paradise. PLoS Biol 16 10.1371/journal.pbio.2006962 PMC6245505 30457985

[CR95] Liu S, Aageaard A, Bechsgaard J, Bilde T (2019) DNA methylation patterns in the social spider, *Stegodyphus dumicola*. Genes. 10. 10.3390/genes10020137 10.3390/genes10020137 PMC6409797 30759892

[CR96] Lubin Y, Bilde T (2007) The evolution of sociality in spiders. In: Advances in the study of behavior, vol 37. Advances in the study of behavior. pp 83-145. 10.1016/s0065-3454(07)37003-4

[CR97] Lubin Y, Birkhofer K, Berger-Tal R, Bilde T (2009). Limited male dispersal in a social spider with extreme inbreeding. Biol J Linnean Soc.

[CR98] Lucchesi JC, Kuroda MI (2015) Dosage compensation in *Drosophila*. Cold Spring Harb Perspect Biol 7:a019398 10.1101/cshperspect.a019398 PMC4448616 25934013

[CR99] Maddison WP, Leduc-Robert G (2013). Multiple origins of sex chromosome fusions correlated with chiasma localization in *Habronattus* jumping spiders (Araneae: Salticidae). Evolution.

[CR100] Mappes J, Alatalo RV, Kotiaho J, Parri S (1996). Viability costs of condition-dependent sexual male display in a drumming wolf spider. Proc R Soc B Biol Sci.

[CR181] Margraf A, Costa-Ayub CLS, Okada MA, Gomes JR, Ortolani-Machado CF, Soares MAM (2011) Development of *Loxosceles intermedia* Mello-Leitão (1934) (Araneae, Sicariidae) genital tract. Brazilian Journal of Biology 71 (3):747–754 10.1590/s1519-69842011000400021 21881800

[CR101] Martišová M, Bilde T, Pekar S (2009). Sex-specific kleptoparasitic foraging in ant-eating spiders. Anim Behav.

[CR102] Mason DA, Rabinowitz JS, Portman DS (2008). dmd-3, a doublesex-related gene regulated by tra-1, governs sex-specific morphogenesis in *C. elegans*. Development..

[CR103] Mathelier A (2016). JASPAR 2016: a major expansion and update of the open-access database of transcription factor binding profiles. Nucleic Acids Res.

[CR104] Matson CK, Murphy MW, Griswold MD, Yoshida S, Bardwell VJ, Zarkower D (2010). The mammalian doublesex homolog DMRT1 is a transcriptional gatekeeper that controls the mitosis versus meiosis decision in male germ cells. Dev Cell.

[CR105] Mestre L, Bonte D (2012). Food stress during juvenile and maternal development shapes natal and breeding dispersal in a spider. Behav Ecol.

[CR106] Michalik P, Uhl G (2005). The male genital system of the cellar spider *Pholcus phalangioides* (Fuesslin, 1775) (Pholcidae, Araneae): development of spermatozoa and seminal secretion. Front Zool.

[CR107] Michalik P, Uhl G (2011). Cephalic modifications in dimorphic dwarf spiders of the genus *Oedothorax* (Erigoninae, Linyphiidae, Araneae) and their evolutionary implications. J Morphol.

[CR187] Mittal OP (1964) Karyological studies on the Indian spiders II. An analysis of the chromosomes during spermatogenesis in five species of spiders belonging to the family Salticidae. Research Bulletin (NS) of the Panjab University 15:315-326

[CR108] Moon M-J, An J-S (2006). Microstructure of the silk apparatus of the comb-footed spider, *Achaearanea tepidariorum* (Araneae: Theridiidae). Entomol Res.

[CR109] Mora G (1990). Paternal care in a neotropical harvestman, *Zygopachylus albomarginis* (Arachnida, Opiliones: Gonyleptidae). Anim Behav.

[CR110] Muyle A, Kafer J, Zemp N, Mousset S, Picard F, Marais GAB (2016). SEX-DETector: a probabilistic approach to study sex chromosomes in non-model organisms. Genome Biol Evol.

[CR111] Muyle A, Shearn R, Marais GAB (2017). The evolution of sex chromosomes and dosage compensation in plants. Genome Biol Evol..

[CR112] Nessler SH, Uhl G, Schneider JM (2007). Genital damage in the orb-web spider *Argiope bruennichi* (Araneae: Araneidae) increases paternity success. Behav Ecol.

[CR113] Neumann R, Ruppel N, Schneider JM (2017) Fitness implications of sex-specific catch-up growth in *Nephila senegalensis*, a spider with extreme reversed SSD. Peerj. 5. 10.7717/peerj.4050 10.7717/peerj.4050 PMC5694211 29158981

[CR114] Oda H, Iwasaki-Yokozawa S, Usui T, Akiyama-Oda Y (2019) Experimental duplication of bilaterian body axes in spider embryos: Holm’s organizer and self-regulation of embryonic fields. Dev Genes Evol:1–15. 10.1007/s00427-019-00631-x 10.1007/s00427-019-00631-x PMC7128006 30972574

[CR115] Otto SP (2009). The evolutionary enigma of sex. Am Nat.

[CR116] Paese CLB, Schoenauer A, Leite DJ, Russell S, McGregor AP (2018) A SoxB gene acts as an anterior gap gene and regulates posterior segment addition in a spider. Elife. 7. 10.7554/eLife.37567 10.7554/eLife.37567 PMC6167052 30126532

[CR117] Pal A, Vicoso B (2015). The X chromosome of Hemipteran insects: conservation, dosage compensation and sex-biased expression. Genome Biol Evol.

[CR118] Palmer DH, Rogers TF, Dean R, Wright AE (2019) How to identify sex chromosomes and their turnover. Mol Ecol. 28:4709-4724 10.1111/mec.15245 PMC6900093 31538682

[CR119] Panara V, Budd GE, Janssen R (2019). Phylogenetic analysis and embryonic expression of panarthropod Dmrt genes. Front Zool.

[CR120] Parker GA, Baker RR, Smith VGF (1972). The origin and evolution of gamete dimorphism and the male-female phenomenon. J Theor Biol.

[CR121] Pätau K (1948). X-segregation and heterochromasy in the spider *Aranea reaumuri*. Heredity..

[CR122] Pechmann M, Khadjeh S, Turetzek N, McGregor AP, Damen WGM, Prpic N-M (2011) Novel function of Distal-less as a gap gene during spider segmentation. PLoS Genet 7. 10.1371/journal.pgen.1002342 10.1371/journal.pgen.1002342 PMC3197691 22028676

[CR123] Pechmann M, Benton MA, Kenny NJ, Posnien N, Roth S (2017) A novel role for Ets4 in axis specification and cell migration in the spider *Parasteatoda tepidariorum*. Elife. 6. 10.7554/eLife.27590 10.7554/eLife.27590 PMC5574703 28849761

[CR124] Pechová (2018) Comparative study of sex chromosomes in spiders. University of South Bohemia

[CR125] Pekár S, Petráková L, Bulbert MW, Whiting MJ, Herberstein ME (2017). The golden mimicry complex uses a wide spectrum of defence to deter a community of predators. Elife..

[CR126] Puzin C, Bonte D, Pétillon J (2019). Influence of individual density and habitat availability on long-distance dispersal in a salt-marsh spider. Ethol Ecol Evol.

[CR127] Quade FSC, Holtzheimer J, Frohn J, Töpperwien M, Salditt T, Prpic N-M (2019). Formation and development of the male copulatory organ in the spider *Parasteatoda tepidariorum* involves a metamorphosis-like process. Sci Rep.

[CR182] Rash LD, King RG, Hodgson WC (2000) Sex differences in the pharmacological activity of venom from the white-tailed spider (*Lampona cylindrata*). Toxicon 38 (8):1111–1127. 10.1016/s0041-0101(99)00226-3 10.1016/s0041-0101(99)00226-3 10708802

[CR128] Revell S (1947). Controlled X-segregation at meiosis in *Tegenaria*. Heredity..

[CR129] Rice WR (1984). Sex chromosomes and the evolution of sexual dimorphism. Evolution..

[CR130] Rice WR (1996). Sexually antagonistic male adaptation triggered by experimental arrest of female evolution. Nature..

[CR131] Robinson MH, Robinson BC (1973). The stabilimenta of *Nephila clavipes* and the origins of stabilimentum-building in araneids. Psyche J Entomol.

[CR132] Roff DA (1992). The evolution of life histories. Theories and analysis.

[CR133] Rovatsos M, Kratochvíl L (2017). Molecular sexing applicable in 4000 species of lizards and snakes? From dream to real possibility. Methods Ecol Evol.

[CR134] Rypstra AL, Schlosser AM, Sutton PL, Persons MH (2009). Multimodal signalling: the relative importance of chemical and visual cues from females to the behaviour of male wolf spiders (Lycosidae). Anim Behav.

[CR135] Salz HK (2011). Sex determination in insects: a binary decision based on alternative splicing. Curr Opin Genet Dev.

[CR136] Samadi L, Schmid A, Eriksson BJ (2015). Differential expression of retinal determination genes in the principal and secondary eyes of *Cupiennius salei* Keyserling (1877). EvoDevo..

[CR137] Sandelin A, Alkema W, Engström P, Wasserman WW, Lenhard B (2004). JASPAR: an open-access database for eukaryotic transcription factor binding profiles. Nucleic Acids Res.

[CR138] Schneider JM (2014) Sexual cannibalism as a manifestation of sexual conflict. Cold Spring Harb Perspect Biol 6. 10.1101/cshperspect.a017731 10.1101/cshperspect.a017731 PMC4413240 25213095

[CR139] Schneider JM, Andrade MCB (2011). Mating behaviour and sexual selection. Spider behaviour: flexibility and versatility.

[CR140] Schneider JM, Fromhage L, Kappeler PI (2010). Monogynous mating strategies in spiders. Behaviour: evolution and mechanisms.

[CR141] Schneider JM, Roos J, Lubin Y, Henschel JR (2001). Dispersal of *Stegodyphus dumicola* (Araneae, Eresidae): they do balloon after all!. J Arachnol.

[CR142] Schomburg C (2017) Developmental studies on eye types and pedipalps in *Parasteatoda tepidariorum*. PhD Thesis, Georg-August-Universiät Göttingen

[CR143] Schomburg C, Turetzek N, Schacht MI, Schneider J, Kirfel P, Prpic N-M, Posnien N (2015). Molecular characterization and embryonic origin of the eyes in the common house spider *Parasteatoda tepidariorum*. EvoDevo..

[CR144] Schönauer A, Paese CL, Hilbrant M, Leite DJ, Schwager EE, Feitosa NM, Eibner C, Damen WG, McGregor A (2016). The Wnt and Delta-Notch signalling pathways interact to direct pair-rule gene expression via caudal during segment addition in the spider *Parasteatoda tepidariorum*. Development..

[CR145] Schulz S, Carde R, Millar J (2004). Semiochemistry of spiders. Advances in chemical ecology.

[CR146] Schulz S, Toft S (1993). Identification of a sex-pheromone from a spider. Science..

[CR147] Schütz D, Taborsky M (2003). Adaptations to an aquatic life may be responsible for the reversed sexual size dimorphism in the water spider, *Argyroneta aquatica*. Evol Ecol Res.

[CR148] Sentenská L, Pekár S (2013). Mate with the young, kill the old: reversed sexual cannibalism and male mate choice in the spider *Micaria sociabilis* (Araneae: Gnaphosidae). Behav Ecol Sociobiol.

[CR149] Sentenská L, Pekár S (2014). Eat or not to eat: reversed sexual cannibalism as a male foraging strategy in the spider *Micaria sociabilis* (Araneae: Gnaphosidae). Ethology..

[CR150] Settepani V, Schou MF, Greve M, Grinsted L, Bechsgaard J, Bilde T (2017). Evolution of sociality in spiders leads to depleted genomic diversity at both population and species levels. Mol Ecol.

[CR151] Sharma A (2017). Male sex in houseflies is determined by Mdmd, a paralog of the generic splice factor gene CWC22. Science..

[CR152] Shen MM, Hodgkin J (1988). mab-3, a gene required for sex-specific yolk protein expression and a male-specific lineage in *C. elegans*. Cell..

[CR153] Smith DR, Su YC, Berger-Tal R, Lubin Y (2016). Population genetic evidence for sex-specific dispersal in an inbred social spider. Ecol Evol.

[CR154] Stavenga DG, Otto JC, Wilts BD (2016) Splendid coloration of the peacock spider *Maratus splendens*. J R Soc Interface 13:20160437 10.1098/rsif.2016.0437 PMC5014068 27512139

[CR155] Stollewerk A, Schoppmeier M, Damen WGM (2003). Involvement of Notch and Delta genes in spider segmentation. Nature..

[CR156] Stratton GE (2005). Evolution of ornamentation and courtship behavior in *Schizocosa* insights from a phylogeny based on morphology (Araneae, Lycosidae). J Arachnol.

[CR185] Suzuki S (1952) Cytological studies in spiders II. Chromosomal investigation in twenty two species of spiders belonging to the four families, Clubionidae, Sparassidae, Thomisidae and Oxyopidae, which constitute Clubionoidea, with special reference to sex chromosomes. J Sci Hiroshima Univ B 13:1-52

[CR157] Suzuki S (1954). Cytological studies in spiders. III. Studies on the chromosomes of fifty-seven species of spiders belonging to seventeen families, with general considerations on chromosomal evolution. J Sci Hiroshima Univ B.

[CR158] Uhl G (2013) Spider olfaction: attracting, detecting, luring and avoiding. In: Nentwig W (ed) Spider ecophysiology. Springer, pp 141–157

[CR159] Uhl G, Elias DO, Herberstein ME (2011). Communication. Spider behaviour: flexibility and versatility.

[CR160] Uhl G, Schmitt S, Schäfer MA, Blanckenhorn W (2004). Food and sex-specific growth strategies in a spider. Evol Ecol Res.

[CR161] Uhl G, Nessler SH, Schneider J (2007). Copulatory mechanism in a sexually cannibalistic spider with genital mutilation (Araneae: Araneidae: *Argiope bruennichi*). Zoology..

[CR162] Uhl G, Nessler SH, Schneider JM (2010). Securing paternity in spiders? A review on occurrence and effects of mating plugs and male genital mutilation. Genetica..

[CR163] Uhl G, Zimmer SM, Renner D, Schneider JM (2015). Exploiting a moment of weakness: male spiders escape sexual cannibalism by copulating with moulting females. Sci Rep.

[CR164] Vanacker D, Vanden Borre J, Jonckheere A, Maes L, Pardo S, Hendrickx F, Maelfait J-P (2003). Dwarf spiders (Erigoninae, Linyphiidae, Araneae): good candidates for evolutionary research. Belgian J Zool.

[CR165] Vanthournout B, Busck MM, Bechsgaard J, Hendrickx F, Schramm A, Bilde T (2018) Male spiders control offspring sex ratio through greater production of female-determining sperm. Proceedings of the Royal Society B: Biological Sciences. 285. 10.1098/rspb.2017.2887 10.1098/rspb.2017.2887 PMC5897641 29563266

[CR166] Vicoso B (2019). Molecular and evolutionary dynamics of animal sex-chromosome turnover. Nat Ecol Evol.

[CR167] Vicoso B, Bachtrog D (2015) Numerous transitions of sex chromosomes in Diptera. PLoS Biol 13 10.1371/journal.pbio.1002078 PMC4400102 25879221

[CR168] Vicoso B, Emerson JJ, Zektser Y, Mahajan S, Bachtrog D (2013) Comparative sex chromosome genomics in snakes: differentiation, evolutionary strata, and lack of global dosage compensation. PLoS Biol 11. 10.1371/journal.pbio.1001643 10.1371/journal.pbio.1001643 PMC3754893 24015111

[CR169] Vöcking O, Uhl G, Michalik P (2013). Sperm dynamics in spiders (Araneae): ultrastructural analysis of the sperm activation process in the garden spider *Argiope bruennichi* (Scopoli, 1772). PLoS One.

[CR170] Vollrath F (1986). Eusociality and extraordinary sex ratios in the spider *Anelosimus eximius* (Araneae: Theridiidae). Behav Ecol Sociobiol.

[CR171] Watson PJ (1986) Transmission of a female sex pheromone thwarted by males in the spider *Linyphia litigiose* (Linyphiidae). Science 219–221 10.1126/science.3726530 3726530

[CR172] Watson PJ (1990). Female-enhanced male competition determines the first mate and principal sire in the spider *Linyphia titigiosa* (Linyphiidae). Behav Ecol Sociobiol.

[CR173] Watson PJ (1991). Multiple paternity and first mate sperm precedence in the sierra dome spider *Linyphia litigiosa* Keyserling (Linyphiidae). Anim Behav.

[CR174] Weyman GS (1993). A review of the possible causative factors and significance of ballooning in spiders. Ethol Ecol Evol.

[CR175] Wickler W, Seibt U (1986). Aerial dispersal by ballooning in adult *Stegodyphus mimosarum*. Die Naturwissenschaften.

[CR176] Wignall AE, Herberstein ME (2013). Male courtship vibrations delay predatory behaviour in female spiders. Sci Rep.

[CR177] Wilhelm D, Palmer S, Koopman P (2007). Sex determination and gonadal development in mammals. Physiol Rev.

[CR178] World Spider Catalogue (2019) Natural History Museum Bern. http://wsc.nmbe.ch. Accessed January 2020

[CR179] Yoshimoto S, Okada E, Umemoto H, Tamura K, Uno Y, Nishida-Umehara C, Matsuda Y, Takamatsu N, Shiba T, Ito M (2008). A W-linked DM-domain gene, DM-W, participates in primary ovary development in *Xenopus laevis*. Proc Natl Acad Sci U S A.

[CR180] Zuk M, Garcia-Gonzalez F, Herberstein ME, Simmons LW (2014). Model systems, taxonomic bias, and sexual selection: beyond *Drosophila*. Annu Rev Entomol.

